# Definition of “persistent vomiting” in current medical literature: A systematic review

**DOI:** 10.1097/MD.0000000000008025

**Published:** 2017-11-10

**Authors:** Mostafa Ebraheem Morra, Abdelrahman Elshafay, Aswin Ratna Kansakar, Ghaleb Muhammad Mehyar, Nguyen Phan Hoang Dang, Omar Mohamed Mattar, Somia Iqtadar, Mostafa Reda Mostafa, Vu Ngoc Hai, Tran Le-Huy Vu, Ahmed Abdelmotaleb Ghazy, Fatima Kaboub, Nguyen Tien Huy, Kenji Hirayama

**Affiliations:** a Faculty of Medicine, Al-Azhar University; b Dirghayu Guru Hospital and Research Center, Chabahil, Kathmandu, Nepal; c Al-Essra Hospital, Amman, Jordan; d Pham Ngoc Thach University of Medicine; e Faculty of Medicine, Cairo University, Cairo; f Faculty of Medicine, King Edward Medical University, Lahore, Pakistan; g Faculty of Medicine, Tanta University, Tanta; h University of Medicine and Pharmacy, District 5; i University of California, Los Angeles, California; j Department of Health Ministry, Kafr El-Zayat, Gharbeya, Egypt; k Faculty of Medicine, University of Mentouri Constantine, Constantine, Algeria; l Evidence Based Medicine Research Group & Faculty of Applied Sciences, Ton Duc Thang University, Ho Chi Minh City, Vietnam; m Department of Clinical Product Development; n Department of Immunogenetics, Institute of Tropical Medicine (NEKKEN), Leading Graduate School Program, and Graduate School of Biomedical Sciences, Nagasaki University, Nagasaki, Japan.

**Keywords:** consensus, persistent vomiting, systematic review

## Abstract

**Background and Aim:**

Persistent vomiting is mentioned as a symptom of a large variety of systemic disorders. It is commonly used interchangeably with chronic, recurrent, or intractable vomiting and widely used as a warning sign of severe illness in dengue infection. However, it has been poorly defined in the medical literature. Therefore, we aimed to systematically review a definition of persistent vomiting in the medical literature.

**Methods:**

A systematic search was done through; PubMed, Google Scholar, Web of Science, Scopus, VHL, WHO-GHL, Grey Literature Report, POPLINE, and SIGLE for the last 10 years. Consensus on the definition was considered to be reached if at least 50% of studies described the same definition using the Delphi consensus technique.

**Result:**

Of 2362 abstracts reviewed, 15 studies were selected based on the inclusion criteria. Three studies used the same definition. Another 2 studies defined it as vomiting of all foods and fluid in 24 hours. Three studies defined persistent vomiting in the units of days or weeks. Four studies used the number of episodes: ≥2 episodes 15 minutes apart, >3 episodes in 12 hours, and >3 episodes within 24 hours.

**Conclusion:**

No consensus for the definition was found among authors. This is a point of concern that needs to be addressed by further studies.

## 1 Introduction

Vomiting or emesis is clinically defined as the oral eviction of gastrointestinal contents, due to contractions of the gut and the muscles of the thoracoabdominal wall.^[1]^ This is somehow different from regurgitation which has been defined as egression of gastric contents to the mouth effortlessly. Retching is the contracting action of the muscles but with no vomitus (i.e., dry heaves). While nausea is spontaneous sensation of the need to vomit.^[2]^ Nausea is not necessarily accompanied by vomiting or retching, and should not be confused/adjoined with dyspepsia, which comprises epigastric burning, gnawing disturbance, bloating, or pain. Physiologically, vomiting is a somatic motor event, which is controlled by the emetic center and chemoreceptor trigger zone of the medulla.^[2]^ Stimulating these centers triggers a series of coordinated motor events to induce vomiting.^[3]^ Indeed, nausea and vomiting are considered reasonable symptoms for patients to seek physical examination.^[4]^

An Australian study showed that about 1.6% (1.5 million) of the medical consultations in a primary care setting per year were for nausea or vomiting.^[5]^ Although nausea and vomiting are common reasons for a consultation in general practice,^[4,6]^ these symptoms are often self-limiting.^[7]^ Vomiting may be worrisome in few situations and, therefore,^[8,9]^ it is important to detect the causes and to eliminate acute emergencies.^[7]^ Through appropriate management of nausea and vomiting, it can constringe the occurrence of hurtful sequel particularly aspiration hazard.^[10]^ In determining the reason for nausea or vomiting, a first consideration is whether the symptoms are persistent, recurrent, acute, or chronic. Persistent vomiting is mentioned as a symptom of a large variety of systemic disorders including; obstruction, gastrointestinal disorders, infectious diseases, neurological disorders, metabolic and endocrine disorders, renal disorders, toxins, postoperation,^[2]^ and pregnancy.^[11]^ It is commonly used interchangeably with chronic, recurrent, or intractable vomiting^[10–14]^ and widely used as a warning sign of severe illness in dengue infection.^[15]^ However, its definition in the medical literature has not been established. Since persistent vomiting has been used as a warning sign of dengue classification, lack of consensus on an appropriate definition may delay diagnosis and management or over-grade the severity of the disease.^[16,17]^ Therefore, we aimed to systematically review the definitions of persistent vomiting in all relevant original studies.

## 2 Materials and methods

### 2.1 Search strategy

The performance of our study follows the recommendation of the PRISMA statement, and our protocol was registered at PROSPERO in September 2015. Our review of literature contains no human participants, therefore ethical approval was not necessary. In August 2015, we conducted a systematic search for studies that define persistent vomiting in 9 electronic databases/search engines, including; PubMed, Google Scholar, Web of Science, Scopus, Virtual Health Library (VHL), World Health Organization-Global Health Library (WHO-GHL), New York Academy of Medicine Grey Literature Report, POPLINE, and System for Information on Grey Literature in Europe (SIGLE). We used the following search terms (“persistent vomiting” OR “persistent emesis”) for all databases, except for Google Scholar, which we searched using a combination of keywords

A. “Persistent vomiting ∗ defined” 2. “Defined ∗ persistent vomiting” 3. “Definition ∗ persistent vomiting” 4. “Persistent vomiting defined” 5. Any word of “persistent vomiting” in title
B. “Persistent emesis ∗ defined” 2. “Defined ∗ persistent emesis” 3. “Definition ∗ persistent emesis” 4. “Persistent emesis defined” 5. Any word of “persistent emesis” in the title.

We excluded animal studies in data extraction and title/abstract screening.

### 2.2 Selection criteria

Two reviewers independently screened the titles and abstracts of the search results for inclusion and exclusion criteria. Any discrepancies were solved by discussion and if required, we consulted a third reviewer. We included any original publication that defined persistent vomiting if it met the following criteria: human participant studies. Articles published after and including the year 2005. No restriction was made with respect to population (age, ethnicity). Exclusion criteria were: animal studies, Overlapped data sets, Articles with only abstracts, Thesis, book, reviews, conference papers, case reports. Articles not published in English, Articles whose full texts were not available and. Articles published before 2005.

We removed duplicates automatically with EndNote Ver. X7 (Thompson Reuter, NY) using the “find duplicates” feature and “author, year, title” as criteria and manually; using the title and abstract screening.

### 2.3 Data extraction

A standardized data extraction form was built up on a pilot extraction of 2 selected references and comprised of 3 components: study setting and design, the definition of persistent vomiting, and methodological quality. Three reviewers independently extracted the data. When there was a disagreement in any information retrieved, a discussion among 3 reviewers was held to find a consensus. If the 3 reviewers could not reach an agreement, the supervisors (NTH, KH) were consulted.

### 2.4 Data analysis

The definition used for persistent vomiting was described in terms of a number of episodes of vomiting and the duration of vomiting. We used a Delphi consensus method to define the persistent vomiting.^[18]^ Consensus on the definition of persistent vomiting was considered to be reached if at least 50% of studies used the same definition.^[19]^

## 3 Results

### 3.1 Search results and study characteristics

**Figure 1:**
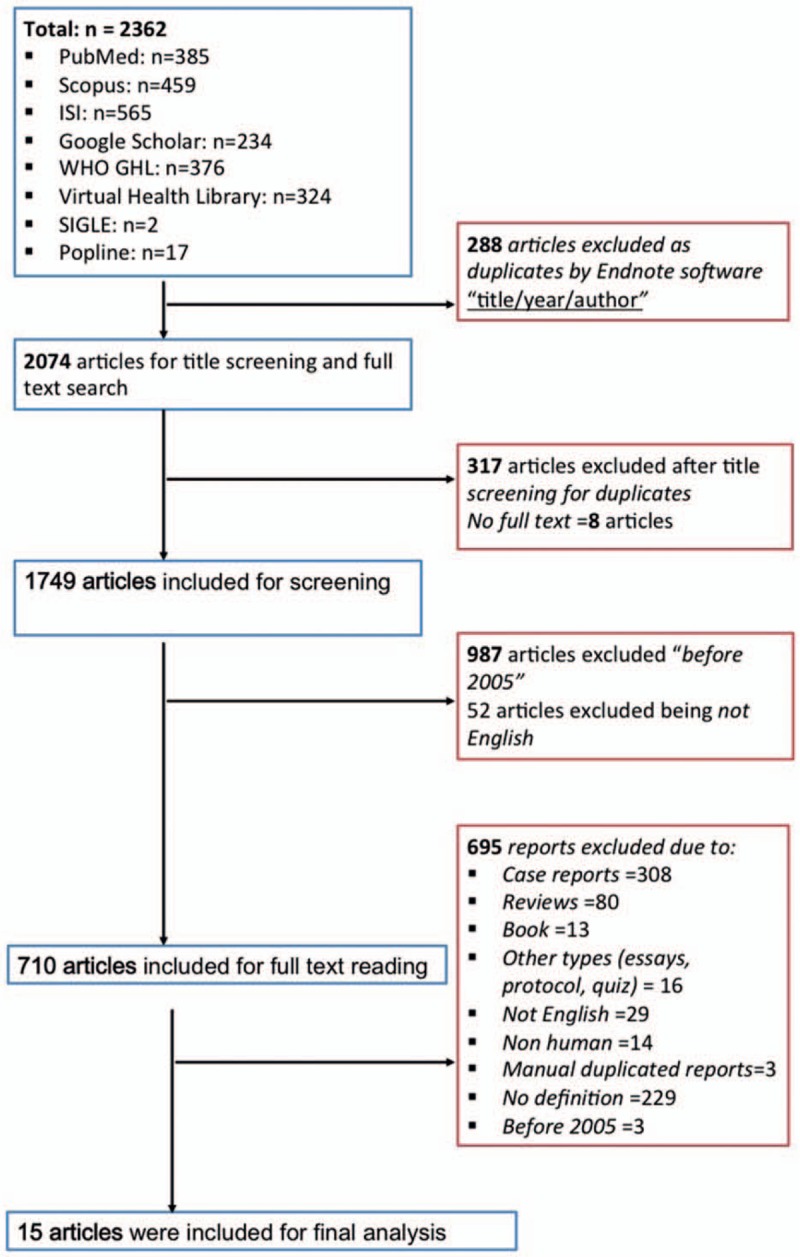
Summary of how the systematic search was conducted and eligible studies were identified (PRISMA flow diagram). PRISMA = Preferred Reporting Items for Systematic reviews and Meta-Analyses.

The total number of references identified by using our search strategy was 2362. After duplicates deletion with Endnote X7 (Thomson Reuters) and title and abstract screening with the aforementioned criteria, there were 15 references for final analysis (Fig. 1). The characteristics of included studies are shown in Table 1. These studies were conducted in Asia (n = 5),^[20–24]^ Africa (n = 5),^[20,24–27]^ Europe (n = 6),^[21,22,27–30]^ North America (n = 8),^[25,27,28,30–33]^ and South America (n = 1).^[32]^ Since persistent vomiting can be a clinical manifestation associated with a number of diseases, we included studies from almost all systems of the body, including respiratory tract,^[18,26]^ the gastrointestinal system,^[22,23,25,30,31,34]^ central nervous system,^[33]^ systemic and non-specific infection.^[21,26,29]^ These studies involved subjects from all age groups. Six of the studies^[20,21,27,28,30,31]^ were performed in infants and neonates. Out of these, 3 studies^[21,27,28]^ compared the effects of different antibiotics on infection by dividing the patient population into various groups according to the antibiotic used. Van den Ende et al,^[30]^ used data from 2 centers to compare the surgical outcome between them. Another 5 studies^[23,25,32–34]^ were performed in children within which 1 study^[25]^ compared 2 treatment strategies for children with acute gastroenteritis. The rest of the included studies^[22,24,26,29]^ conducted in adults were about miscellaneous medical disorders. The patient characteristics and the mortalities are reported in Table 1.

**Table 1:**
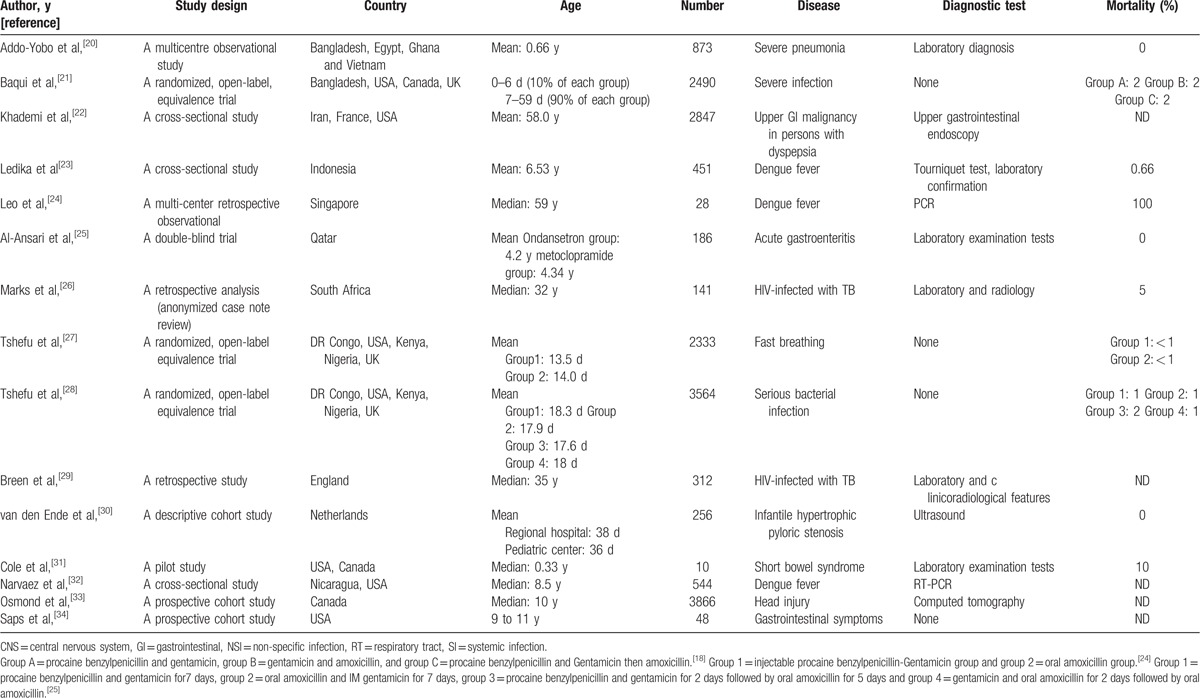
Descriptive characteristics of included studies.

### 3.2 Definition of persistent vomiting

The definition of persistent vomiting in each included study was extracted and is presented in Table 2. The definition of persistent vomiting was defined variably across all studies. As a medical term, persistent vomiting is well known. However, there was no definite definition or clinical criteria to describe it. Among the included studies, some of them described it as the frequency and time of day it occured or about the content of it. Related to a specific condition, persistent vomiting was defined variably in dengue fever.^[24,32,35]^ On the other hand, Marks et al,^[26]^ and Breen et al,^[29]^ used the same definition in the adverse effect of anti-tuberculosis treatment. Moreover, persistent vomiting was defined similarly in adverse effect of antibiotic treatment.^[20,21,28]^ Furthermore, 3 studies^[21,27,28]^ (20%) used the same definition of persistent vomiting describing it as “vomiting after 3 attempts of feeding the baby within 30 minutes”, out of which 2 were conducted by the same authors.^[27,28]^ Another 2 studies^[26,29]^ (13%) defined it as vomiting of all foods and fluid in 24 hours. Four studies (20%) defined persistent vomiting in the units of days^[22,24,30]^ or weeks.^[34]^ Four studies (27%) used the number of episodes in a particular time period as criteria for persistent vomiting. It was defined as ≥2 episodes 15 minutes apart,^[33]^ >3 episodes in 12 hours,^[31,32]^ and >3 episodes within 24 hours.^[25]^

**Table 2:**
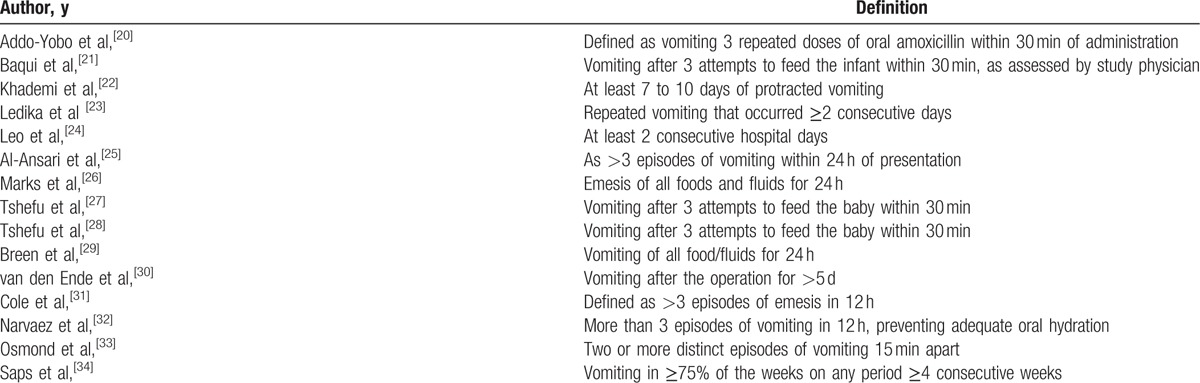
Definitions of persistent vomiting among our included studies.

## 4 Discussion

Persistent vomiting, a frequently used medical term in literature, is to date, a not well-defined characteristic. Our systematic review was carried out to look for any standardized definition of persistent vomiting; keeping in mind the Delphi consensus method that if 50% of the results mentions the same definition a consensus about the definition can be made.^[18]^ All the included studies had a different perspective of defining this symptom which was sometimes measured in terms of a number of episodes and at another instance, time bound ranging from few minutes to even weeks.^[27,32,34]^ The time range is too broad to give even a generalized idea to define it and thus lacks specificity and is of no help in assessing the severity of the symptoms enough to call it as persistent. Also, the contents of the vomitus are also not well defined with few mentioning it as all food taken and others as fluid contents.^[26,29]^ Authors also did not take into account the adult and pediatric population, so the definition of persistent vomiting was vaguely defined in the included studies.

Administration of drugs or food preceding emesis is mentioned in a few studies and time taken from administration to expulsion of food were taken as criteria to define vomiting. However, this lacks standardization as well and is different in babies and in adults.^[20,21,27]^ Also, it is worth considering that the median age of the included studies is a very wide range (0.33–59 years) thus making it more difficult to extract a standardized definition. In children, failed attempts to feed were also taken as a criterion to define persistent vomiting, but the number of attempts was variable and so was the time taken from failed attempt to the expulsion of food.^[27,28]^ One author defined it as a postsurgery complication and lacks a definition in other conditions, making it less reliable to be used as a guide.^[30]^ Review of other characteristics of the included studies also show that this symptom is present in a wide variety of clinical conditions related to different systems of the body, which are linked to different mortality indices ranging from 0%^[20,25,30]^ in severe pneumonia, hypertrophic pyloric stenosis, and acute gastroenteritis to 100%^[24]^ in dengue fever. However, it is inconclusive from the studies to which extent persistent vomiting is related to disease severity and hence morbidity and mortality.

Lack of standard criteria to clearly define persistent vomiting raises the question of the use of this term as a sign of severity and as a marker of some diseases already described in medical literature. This systematic review brings the attention of the medical and research scholars to the need to more precisely define clinical signs and symptoms used in clinical studies to avoid misinterpretation of the data used for research purposes.

In this systematic review, we faced several limitations. One of them was that articles published after 2005 were reviewed; to get more recent findings, however, this led to a small number (n = 15) of included studies, which may have limited the validity of the results. Another limitation is that we could not perform a subgroup analysis for children/adult, infectious/non-infectious, acute/chronic patients because of the small number of included studies. Restricting our search to English articles may have missed some data but this was adopted to minimize any mistranslation from another language which could have affected the results.

## 5 Conclusions

Variable definitions exist in the medical literature to define persistent vomiting with no consensus among authors and is a point of concern that needs to be addressed by further studies.

## Acknowledgments

The authors thank Mahmoud Atef Morsi (Faculty of Medicine, Menoufia University, Shibin El-kom, Egypt), Omar Abdul Bagi Omar (Faculty of Medicine, University of Gezira, Sudan), Ali Mohamed Hammad (Faculty of Medicine, Cairo University, Cairo, Egypt), and Ngo Thi Huyen (University of Medicine and Pharmacy, Ho Chi Minh city, Vietnam) for their contribution in full text finding and screening.
